# A Case of Prolapsed Gastropathy Syndrome Presenting as Hematemesis and Acute Blood Loss Anemia

**DOI:** 10.7759/cureus.12563

**Published:** 2021-01-07

**Authors:** Grace D Hopp, Landon Brown, Tamneet Singh, Allan Parker

**Affiliations:** 1 Internal Medicine, University of Texas Health Science Center at San Antonio, San Antonio, USA; 2 Gastroenterology, University of Texas Health Science Center at San Antonio, San Antonio, USA

**Keywords:** hematemesis, prolapsed gastropathy syndrome, endoscopy, retching

## Abstract

A 45-year-old male with hypertension and alcohol use disorder presented to the hospital after being found intoxicated, with bright red blood in the toilet and around his mouth. He was found to be tachycardiac and required intubation due to his inebriated state to establish a secure airway. Initial workup revealed a hemoglobin decrease from 16.7 g/dL to 8.7 g/dL, as well as lactic acidosis. He quickly underwent an upper endoscopy to evaluate his source of hematemesis. An actively bleeding lesion was found in the proximal stomach consistent with prolapse gastropathy syndrome. This case highlights a unique presentation of hematemesis that requires endoscopic evaluation for both diagnosis and treatment.

## Introduction

Prolapsed gastropathy syndrome is an uncommon cause of hematemesis, seen most commonly with recurrent retching. This is a clinical syndrome defined by the invagination of the gastric mucosa into the lower esophagus. On endoscopy, there is a characteristic well-demarcated hyperemic mucosa. Rarely, after repeated invagination, the stomach mucosa can become hemorrhagic, resulting in upper gastrointestinal bleeding [1]. We present a case of prolapsed gastropathy syndrome in a 45-year-old male with acute alcohol intoxication who presented with hematemesis and anemia due to acute blood loss. 

## Case presentation

A 45-year-old male with hypertension and alcohol use disorder presented to the hospital after he was found intoxicated with bright red blood in the toilet and around his mouth. He was altered on arrival secondary to his inebriated state. On exam, his initial blood pressure was 142/108, heart rate in the 120s, with a Glasgow coma scale (GCS) score of 7. Due to his GCS, he was intubated for airway protection and admitted to the intensive care unit (ICU) for further treatment of his presumed upper gastrointestinal bleed. Initial laboratory results were notable for hemoglobin of 16.7 g/dL, leukocytosis of 12.5 k/mcL, lactic acid of 4.7 mmol/L, aspartate aminotransferase (AST) 73 U/L, alanine aminotransferase (ALT) 68 U/L, gamma-glutamyl transferase (GGT) 418 U/L, and serum ethanol of 454 mg/dL (Table 1). Twenty-four hours later, his hemoglobin had dropped to 8.7 g/dL, without further evidence of hematemesis or melena. 

**Table 1:** Patient's laboratory values with corresponding reference ranges

Laboratory	Patient’s Value	Reference Range	Units
Hemoglobin	16.7	12.8-17.1	g/dL
White Blood Cell Count	12.5	3.40-10.40	k/mcL
Lactic Acid	4.7	0.5 – 2.2	mmol/L
Aspartate Aminotransferase	73	< 35	U/L
Alanine Aminotransferase	68	< 46	U/L
Gamma-Glutamyl Transferase	418	< 178	U/L
Serum Ethanol	454	< 10	g/dL

**Figure 1:**
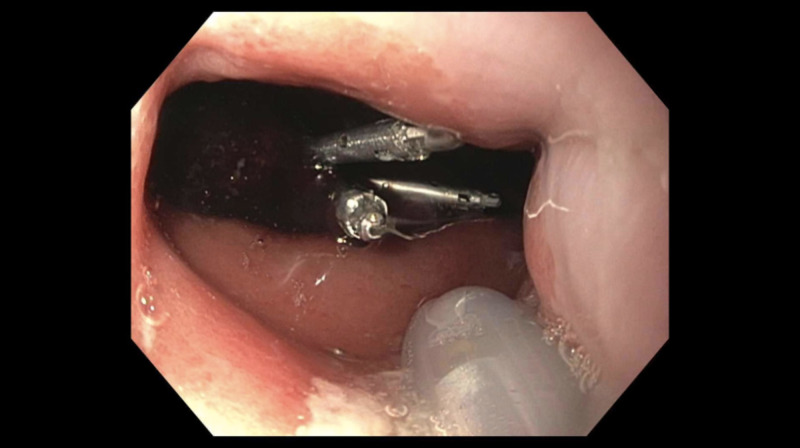
Endoscopic view from the gastroesophageal junction demonstrating adherent clot with three hemoclips in place.

The patient underwent an upper endoscopy, which identified hemorrhagic mucosa in the proximal stomach several centimeters distal from the gastroesophageal junction. The lesion had an adherent clot with actively bleeding at the time of endoscopy. As a result, three hemoclips were placed with adequate hemostasis (Figure 1). The patient was maintained on intravenous proton pump inhibitor twice daily for the remainder of his hospital stay. His hemoglobin recovered to his baseline and he was discharged with plans for a repeat upper endoscopy in eight weeks.

## Discussion

Prolapse gastropathy is a clinical syndrome in which a portion of the gastric mucosa intussuscepts into the lower esophageal sphincter with continuous retching. If the patient retches during endoscopy, the prolapsing mucosa may be visualized in the distal esophagus. More often, the consequences of the continuous prolapsing can be seen at the time of endoscopy. Characteristically, there is a small area of mucosa that appears hyperemic on direct visualization, located in the proximal stomach a few centimeters from the gastroesophageal junction [1]. The mechanism of injury is direct trauma to the mucosa caused by the repeated invagination or incarceration. Patients commonly present with epigastric pain, but may have hematemesis due to severe damage of the mucosa, as in our patient [2].

During our patient’s endoscopic evaluation, there were no other lesions identified that would have led to hematemesis or his acute blood loss anemia. Most likely, his acute intoxication had caused repeated retching prior to presentation. However, due to his inebriated state, he was unable to provide this history. In a case series published by Young et al., three patients who presented with hematemesis in the context of alcohol abuse were noted to have prolapsed gastropathy and only one was noted to have a concomitant Mallory Weiss tear [3].

There was a retrospective study performed by Byfield et al. that reviewed 475 cases of hematemesis [4]. Only seven of those patients (2%) were found to have gastric mucosal prolapse as the cause of their bleeding. Each of the seven patients experienced a variable period of retching and vomiting with subsequent hematemesis. In their discussion, the authors note that each of their patients had an isolated finding of a red congested patch of mucosa in the proximal stomach. The rest of the endoscopic examination of the stomach was unremarkable [4]. This is very similar to the patient we present above. 

The main pillar of treatment for the syndrome is to address the underlying etiology of retching or emesis. In the interim, anti-emetics are used. Most cases of prolapsed gastropathy syndrome are self-limited. If the characteristic lesion is seen without active oozing, there are no endoscopic treatment recommendations.

Prolapse gastropathy syndrome is likely under-reported. This syndrome is important to recognize because it is a significant cause of hematemesis and, like in our patient, may necessitate an ICU stay. This case highlights an atypical cause of gastrointestinal bleeding requiring endoscopic visualization to be recognized and identified.

## Conclusions

Prolapse gastropathy syndrome is characterized by repeated retching, causing invagination of the gastric mucosa into the lower esophageal sphincter. It most often presents as abdominal pain and rarely, hematemesis. Endoscopy reveals an area of hyperemic mucosa just distal to the gastroesophageal junction. It is important to recognize prolapse gastropathy syndrome as it can be a significant cause of hematemesis and resultant blood loss anemia. 
